# Supplementary material for: Topological magneto-optical Kerr effect without spin-orbit coupling in spin-compensated antiferromagnet
Source: Nat Commun. 2026 Mar 3;17:3386. doi: 10.1038/s41467-026-70238-0 (PMC13065855; doi:10.1038/s41467-026-70238-0)
Supplement: Supplementary file 1 — Supplementary Information [file 41467_2026_70238_MOESM1_ESM.pdf]

Supplementary Information for

Topological Magneto-optical Kerr Effect without Spin-orbit Coupling in Spin-compensated Antiferromagnet

Camron Farhang,[1,+] Weihang Lu,[1,+] Kai Du,[2] Yunpeng Gao,[3] Junjie Yang,[3] Sang-Wook Cheong,[2] and Jing Xia[1,*]

[1] Department of Physics and Astronomy, University of California, Irvine, Irvine, CA 92697, USA

[2] Keck Center for Quantum Magnetism and Department of Physics and Astronomy, Rutgers University, Piscataway, NJ 08854, USA.

[3] Department of Physics, New Jersey Institute of Technology, Newark, New Jersey 07102, USA

+These authors contributed equally.

*Correspondence: xia.jing@uci.edu

Contents:

Supplementary figures 1 of extended data	pages 2
Supplementary figures 2 of extended data	pages 3
Supplementary figures 3 of extended data	pages 4
Supplementary figures 4 of extended data	pages 5
Sagnac interferometer for MOKE measurements	page 6
Sagnac interferometer's 55 dB rejection of optical anisotropy	page 7
MOKE after Zero-field Cool (ZFC)	page 9
Magnetic Force Microscopy (MFM)	page 10
Energy-dispersive X-ray spectroscopy (EDX)	page 11
Estimating the Contribution of Magnetization to the MOKE Signal	page 13

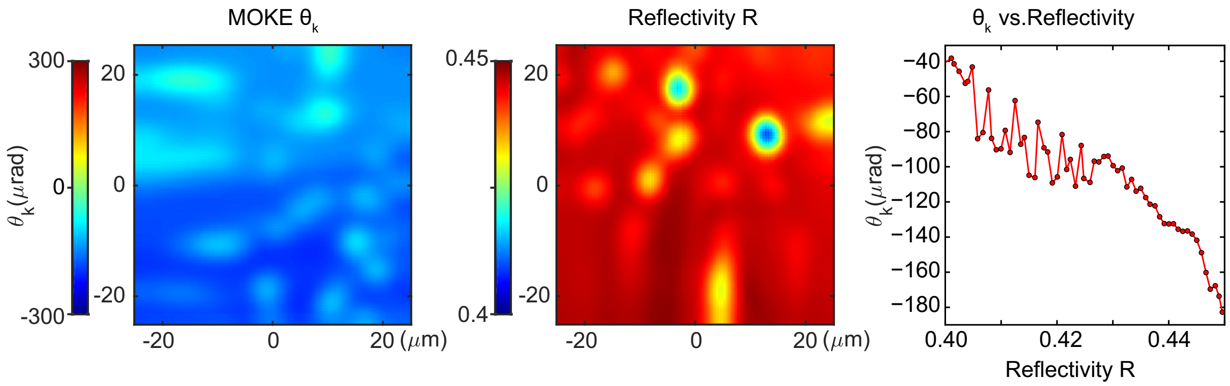

Supplementary Figure 1. Extended data: Imaging the correlation between Co composition and magnetism at 10 K. Spontaneous MOKE image (left), reflectivity image (middle), and their empirical correlation (right).

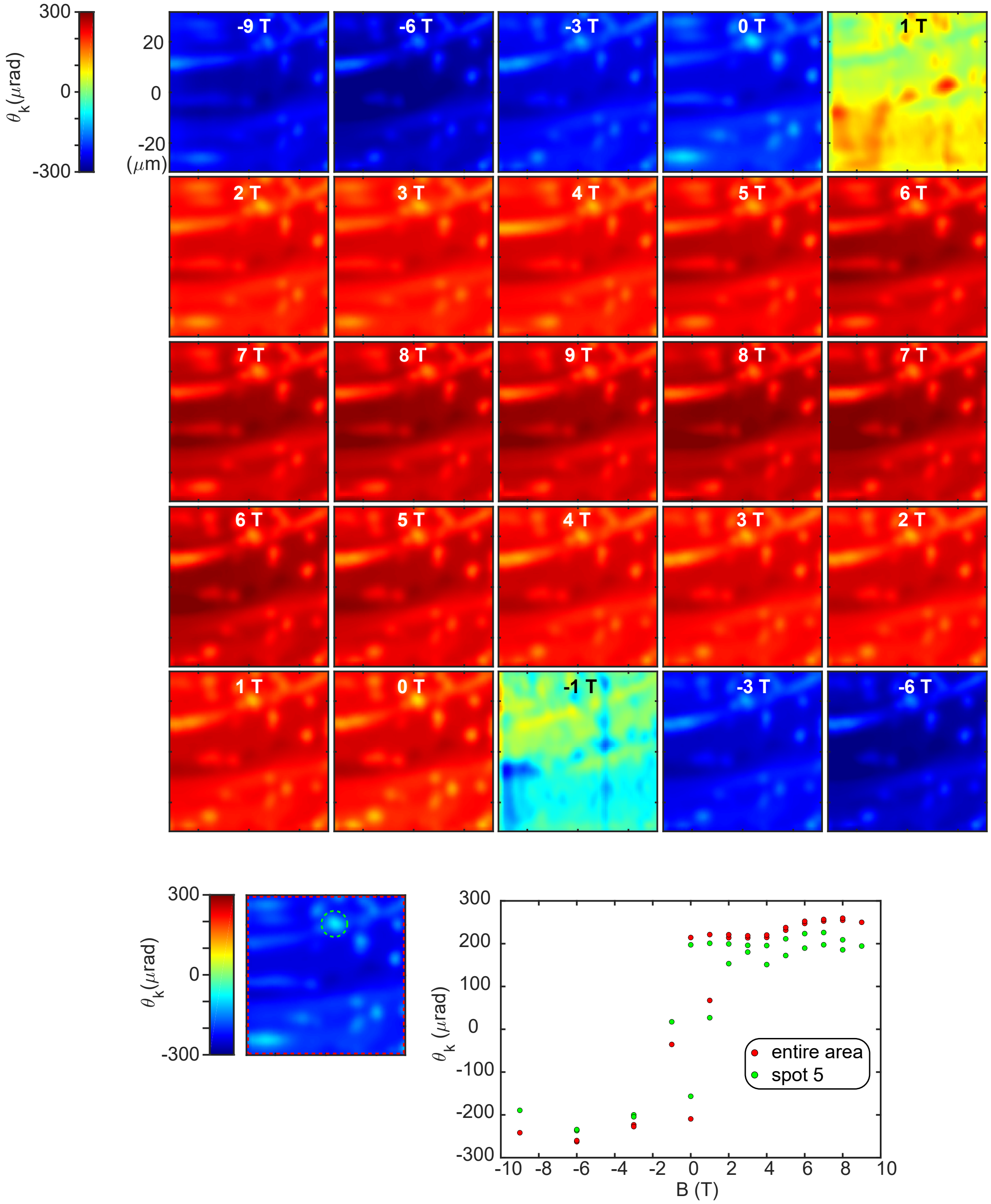

Supplementary Figure 2. Extended data: Chirality domain switching in another region at 10*K*. Top: MOKE images taken during hysteresis at $B = 1T$ interval. Bottom: extracted hysteresis loop from the whole area average and from the circled spot 5.

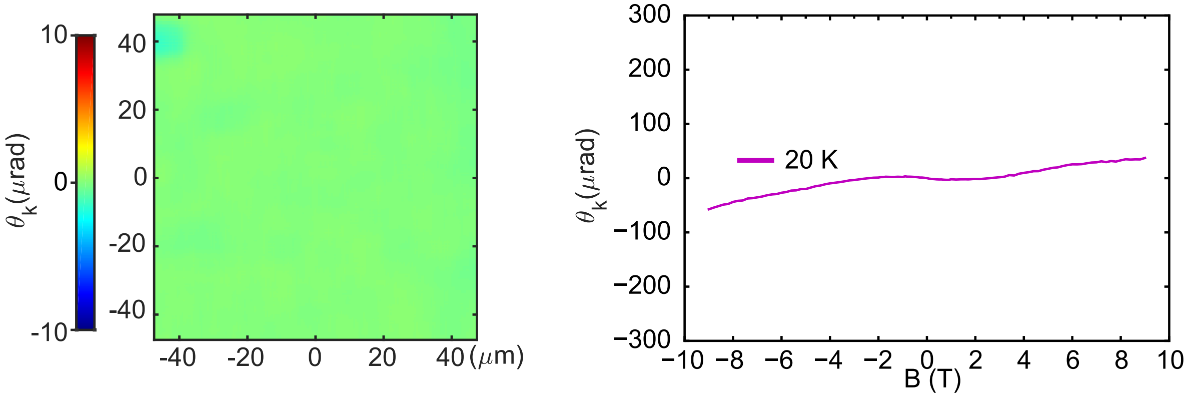

Supplementary Figure 3. Extended data: Zero-field MOKE imaging and hysteresis at 20 *K* in the single-Q phase. No spontaneous θ_K is present in either MOKE image or single-point hysteresis.

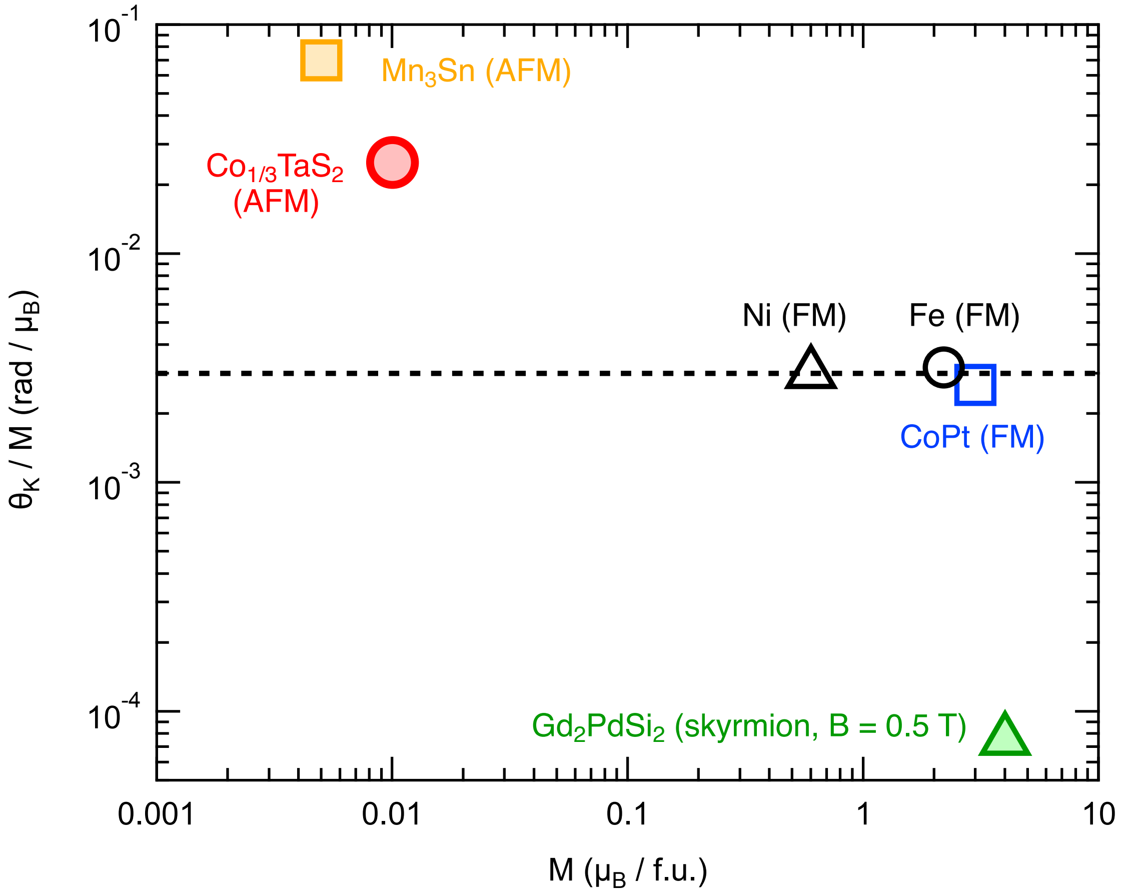

Supplementary Figure 4. Extended data: MOKE over Magnetization ratio ($\boldsymbol{\theta_K/M}$) of representative ferromagnets (FM), antiferromagnets (AFM), and skyrmion lattice. Ferromagnets (Ni [1], Fe [1], and CoPt [2]) have spontaneous θ_K roughly proportional to the magnetization, $\sim 0.03\, rad/\mu_B$. Noncoplanar antiferromagnet $Co_{1/3}TaS_2$ and coplanar antiferromagnet Mn_3Sn [3] have negligible net moments and are significantly above this ratio. Skyrmion lattice Gd_2PdSi_2 [4] is established only within a narrow magnetic field range near $B = 0.5\, T$ and is way below this ratio.

Sagnac interferometer for MOKE measurements

The schematics of the zero-loop Sagnac interferometer [5] used in this work is shown in Supplementary Fig. 5. The beam of light from a CW light source centered at 1550 nm is routed by a fiber-circulator to a fiber-polarizer, which polarizes the beam.

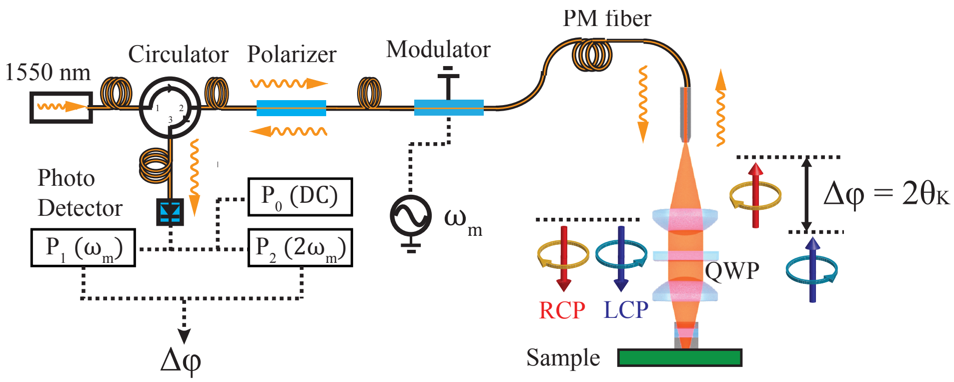

Supplementary Figure 5. Sagnac MOKE setups operating with continuous-wave (CW) light at 1550 nm wavelength: Schematics of a zero-area-loop fiber-optic interferometer that is only sensitive to TRSB (MOKE θ_K) effects, which is independent of α. The fiber-optic head can be scanned to simultaneously acquire reflection and MOKE images.

The circulator transmits light from port 1 to port 2 and from port 2 to port 3 with better than 30 dB isolation in the reverse directions. After the polarizer the polarization of the beam is at 45° to the axis of a fiber-coupled electro-optic modulator (EOM), which generates 4.6 MHz time-varying phase shifts $\phi_m \sin(\omega t)$, where the amplitude $\phi_m = 0.92$ rad between the two orthogonal polarizations that are then launched into the fast and slow axes of a polarization maintaining (PM) single mode fiber. Upon exiting the fiber, the two orthogonally polarized linearly polarized beams are converted into right- and left-circularly polarizations by a quarter-wave plate (QWP) and are then focused through the optical window of the cryostat onto the sample. After reflection from the sample and passing through the optical window, the same quarter-wave plate converts the reflected beams back into linear polarization with exchanged polarization axes. The two beams then pass through the PM fiber and EOM but with exchanged polarization modes in the fiber and the EOM. At this point, the two beams have gone through the same path but in opposite directions, except for a phase difference of $\Delta\varphi$ from reflection off the magnetic sample and another time-varying phase difference by the modulation of EOM. This nonreciprocal phase shift $\Delta\varphi$ between the two counterpropagating circularly polarized beams upon reflection from the sample is twice the Kerr

rotation $\Delta\varphi = 2\theta_K$. The two beams are once again combined at the detector and interfere to produce an optical signal $P(t)$:

$$P(t) = \frac{1}{2} P[1 + \cos(\Delta\varphi + \phi_m \sin(\omega t))] \tag{1}$$

, where P is the returned power if the modulation by the EOM is turned off. For MOKE signals that are slower that the 4.590 MHz modulation frequency used in this experiment, we can treat $\Delta\varphi$ as a slowly time-varying quantity. And $P(t)$ can be further expanded into Fourier series with the first few orders listed below:

$$\begin{aligned} P(t)/P = & \frac{1}{2} [1 + \mathrm{J}_0(2\phi_m)] \\ & +(\sin(\Delta\varphi) J_1(2\phi_m)) \sin(\omega t) \\ & +(\cos(\Delta\varphi) J_2(2\phi_m)) \cos(2\omega t) \\ & +2\, \mathrm{J}_3(2\phi_m)) \sin(3\omega t) \\ & + \cdots \end{aligned} \tag{2}$$

, where $J_1(2\phi_m)$ and $J_2(2\phi_m)$ are Bessel J-functions. Lock-in detection was used to measure the first three Fourier components: the average (DC) power (P0), the first harmonics (P1), and the second harmonics (P2). And the Kerr rotation can then be extracted using the following formula:

$$\theta_K = \frac{1}{2} \Delta\varphi = \frac{1}{2} \tan^{-1}\left[\frac{\mathrm{J}_2(2\phi_m) P_1}{\mathrm{J}_1(2\phi_m) P_2}\right] \tag{3}$$

The noise in Kerr signal is shot-noise-limited to $10^{-7} rad/\sqrt{Hz}$ with 10 μW of optical power, which is small enough not to heat up the sample even at the base temperature of the cryostat. By averaging over 100 seconds, 10 nanoradian (nrad) Kerr resolution can be achieved over a few Kelvins variation of sample temperatures. In practice, the bias offset in our system drifts about 20 nrad in experiments that take a long time or over wide sample temperature ranges. And the flexible fiber head can be mechanically scanned to simultaneously produce reflection (P0) and MOKE (θ_K) images.

Sagnac interferometer's 55 dB rejection of optical anisotropy

An important feature of the Sagnac interferometer for this work is its exclusive detection of microscopic time-reversal symmetry breaking (TRSB) while rejecting non-TRSB effects such as optical birefringence with 55 dB (3×10^{-6}) level of rejection. This is particularly important in $Co_{1/3}TaS_2$ where we have recently discovered a nonvolatile nematic order with a birefringent polarization rotation of $\theta_T = 600\,\mu rad\, sin(2\alpha)$, α being the incident polarization angle [6]. With the 55 dB rejection, we expect the

nematic order will introduce at most 0.002 μrad false signal in the Sagnac measurements, which is below our sensitivity. This selective sensitivity to TRSB effects is achieved by using a single-mode optical fiber as both the source and detector for counterpropagating, time-reversed light beams. According to Onsager's relations, this configuration guarantees zero signal in the absence of TRSB.

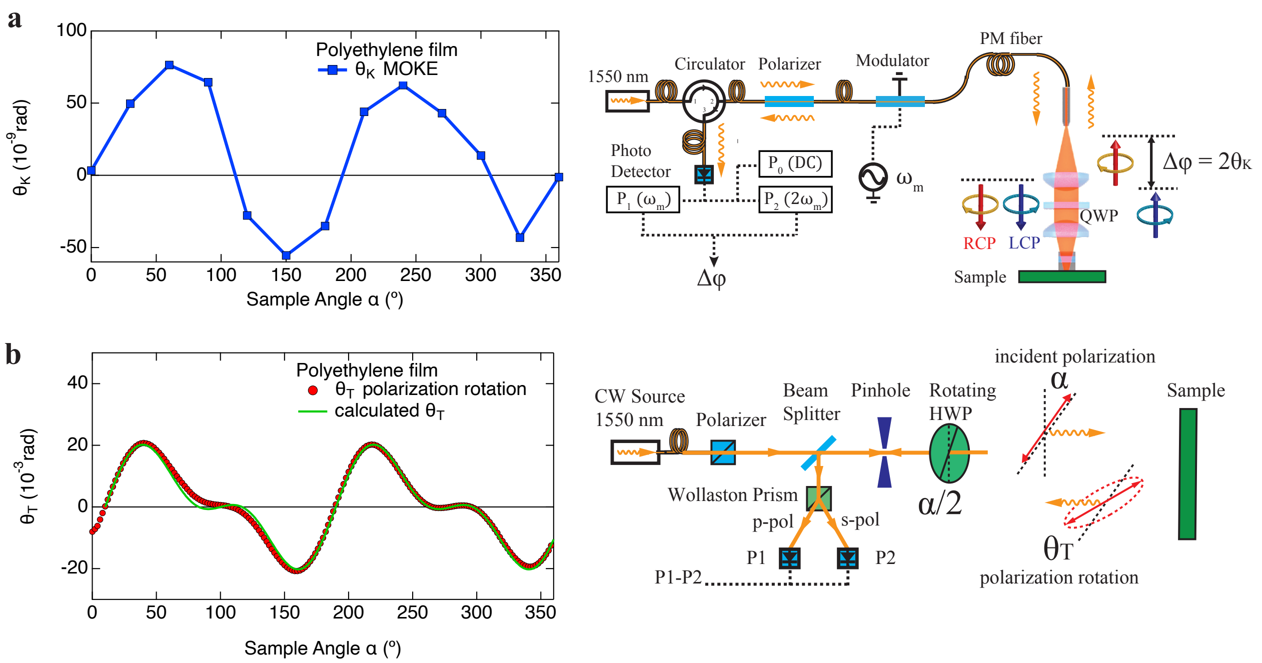

Supplementary Figure 6. 55 dB rejection of optical anisotropy demonstrated in an anisotropic polyethylene film. MOKE θ_K is expected to be zero due to lack of TRSB, while polarization rotation θ_T is expected in this anisotropy film due to optical linear birefringence (LB) and optical linear dichroism (LD). **(a)** Measured θ_K by Sagnac interferometry is smaller than 60 nrad. **(b)** Measured θ_T shows a pattern of 20 mrad that clearly demonstrates an anisotropic (rotational symmetry breaking). Solid line is the calculated polarization rotation for an anisotropic reflective sample, and it agrees well with the measured θ_T. Insets are schematics of Sagnac and polarization rotation measurement setups. Sample angle α is changed by sample rotation with the Sagnac MOKE setup, and by rotating the half-wave plate by $\alpha/2$ in the polarization setup.

The 55 dB rejection of optical anisotropy is demonstrated with a polyethylene film. This polymer film is optically anisotropic and thus produces anisotropic optical rotations. The results are shown in Supplementary Fig. 6. The measured MOKE signal $\theta_K(\alpha)$ (Supplementary Fig. 6a) remains close to zero

($< 60\ nrad$) as the sample doesn't break time-reversal symmetry, while the measured polarization rotation $\theta_T(\alpha)$ (Supplementary Fig. 6b) up to $\pm 20\ mrad$ displays 2-fold rotational symmetry.

The shape of $\theta_T(\alpha)$ in Supplementary Fig. 6b is a direct result of the presence of optical linear birefringence (LB) and optical linear dichroism (LD). And it can be calculated analytically:

$$\theta_T(\alpha) = sin(2\,\alpha)cos(2\,\alpha)\ \frac{e^{i\,2\,LB}\left((LD-2)\,LD\,(2+(LD-2)\,LD)\,e^{i\,2\,LB} + (-(LD-1)^2 - e^{i\,4\,LB}\ (LD-1)^2 + e^{i\,2\,LB}(2+(LD-2)\,LD\,(2+(LD-2)\,LD)))\right)}{2+(LD-2)\,LD\,(2+(LD-2)\,LD)+(-2+LD)\,LD\,(2+(\,LD-2)\,LD)\,cos(2\,\alpha))}$$

The calculated $\theta_T(\alpha)$ curve in Supplementary Fig. 6b is obtained using fitting parameters LB = 0.133 and LD = 0.015, which matches well to the experimental data. This polymer film serves as an example that while the polarization rotation setup detects the total polarization rotation, the Sagnac interferometer is sensitive only to TRSB effects.

MOKE after Zero-field Cool (ZFC)

In addition to the data presented in Fig. 1d, we performed field-cooling (FC) and zero-field-cooling (ZFC) measurements at a different sample location, as shown in Supplementary Fig. 7. Both the 0.3 T FC (blue) and subsequent zero-field warm (ZFW, red) traces in Supplementary Fig. 7a exhibit a large θ_K of 180 μrad, comparable to the 250 μrad signal in Fig. 1d from another location. During FC, a slowly varying background arises from the Faraday effect in optical components located inside the magnetic field, as described in Ref.[7]; this background is absent when no external field is applied. The spin chirality domains are aligned by the magnetic field during cooling, resulting in large MOKE signals both in FC and subsequent ZFW.

In contrast, the ZFC measurement (blue in Supplementary Fig. 7b) yields a much smaller θ_K of $-2\ \mu rad$. Without an applied field during cooling, the chirality domains remain randomly oriented, leading to near-complete cancellation of contributions within the optical probe area.

This contrasting behavior confirms that the MOKE signal originates from the chirality of AFM domains rather than uncompensated moments at AFM domain walls. If the latter were dominant, ZFC that has more AFM domains would produce a larger MOKE signal, contrary to our observations.

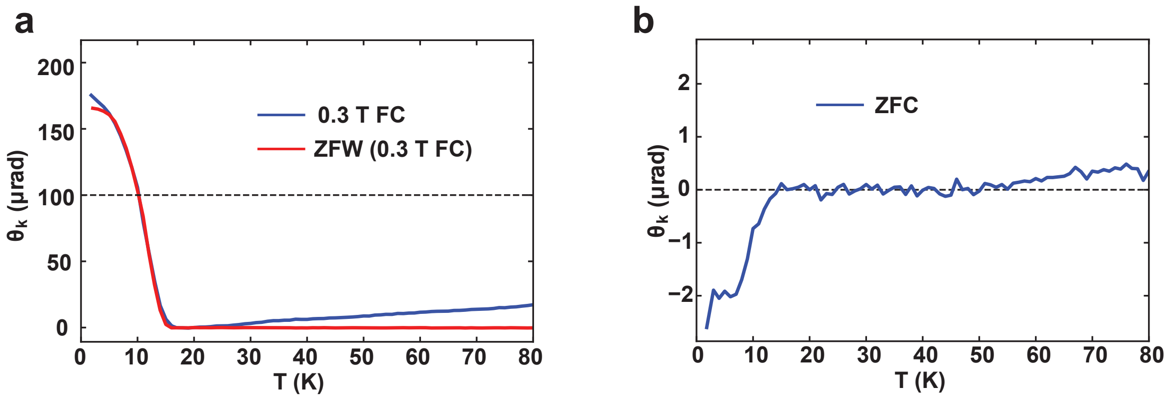

Supplementary Figure 7. Temperature-dependence of MOKE after field-cool (FC) and zero-field-cool (ZFC) at the same location. (a) MOKE θ_K measured during 0.3 T FC (blue) and a subsequent ZFW (red). In the FC (blue), a slowly-temperature-varying contribution is present due to the Faraday effect from optical components inside the magnetic field, which is described in detail in Ref.[7]. Note that such contribution is absent if there is no magnetic field. The spin chirality domains are aligned by the magnetic field during cooling, resulting in large MOKE signal. **(b)** MOKE θ_K measured during zero-field-cool (ZFC) (blue). The spin chirality domains are randomized, resulting in much smaller MOKE signals due to cancellation from oppositely oriented chirality domains within the optical probe beam.

Magnetic Force Microscopy (MFM)

We have conducted extensive low-temperature MFM measurements under various experimental conditions. Supplementary Fig. 8 shows a representative scan taken at 5 K after zero-field cooling, where AFM domains are expected to be most abundant. However, no magnetic domains or domain walls were detected within the MFM sensitivity. This indicates that the fringing fields produced by uncompensated moments at AFM domain walls are too weak to be resolved by MFM. This is consistent with the picture that the MOKE signal is (dominantly) due to the chirality of AFM domains instead of uncompensated magnetic moments at AFM domain walls.

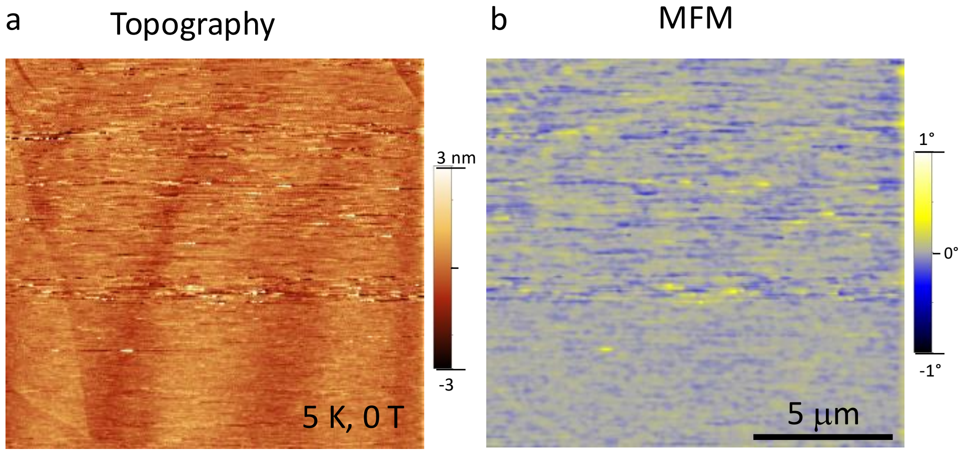

Supplementary Figure 8. Magnetic Force Microscopy (MFM): Low-temperature MFM. Topography **(a)** and MFM phase image **(b)** of cleaved $Co_{1/3}TaS_2$ at 5 K after zero-field cooling. No magnetic domains or domain walls are observed within the sensitivity of MFM.

Energy-dispersive X-ray spectroscopy (EDX)

As discussed in the main text, it is challenging to perform energy-dispersive X-ray spectroscopy (EDX) chemical mapping in the exact same region examined by low-temperature Sagnac microscopy, which provides both MOKE and reflectivity maps at 1550 nm. Both techniques are time-consuming, and the millimeter-sized crystal surface is largely topographically featureless, making it impractical to revisit the same tens-of-microns-sized region with different instruments.

Since no known optical transitions exist in $Co_{1/3}TaS_2$ between red light (620 - 750 nm) and 1550 nm, we assume that the optical reflectivity at 1550 nm qualitatively follows that at red wavelengths. Based on this assumption, we use a wide-field laboratory microscope to measure red-light reflectivity in the same region where EDX was conducted, and infer the corresponding 1550 nm reflectivity.

Supplementary Fig. 9 demonstrates that regions with lower cobalt concentration exhibit higher optical reflectivity at red wavelengths, which we assume also applies at 1550 nm. We first locate a flat 500 μm -wide region in the electron microscope containing several 40 μm pits used as landmarks (Supplementary Fig. 9a). EDX is then performed within four boxes in a smaller 50 μm subregion, yielding cobalt concentrations x in descending order: box 10 (x~0.292±0.015) > box 8 (x~0.290±0.015) > box 9 (x~0.289±0.015) > box 7 (x~0.286±0.015), as shown in Supplementary Fig. 9c. The sample is subsequently transferred to the optical microscope, where the same 500 μm region is located using the same pits as

landmarks, a process that took days (Supplementary Fig. 9b). The red-light reflectivity map reveals very low reflectivity (blue) in the pits and noticeable reflectivity variations (red-yellow-green) even within otherwise flat regions, indicating that the contrast is not purely topographical. Using the pits as reference points, we then image the same 50 μm subregion previously used for EDX (Supplementary Fig. 9b). The mean reflectivity R values follow the inverse trend of Co concentration: box 10 (R~0.31) ≈ box 8 (R~0.31) < box 9 (R~0.33) ≈ box 7 (R~0.33). This correlation supports the picture that higher Co content leads to lower red-light reflectivity, an effect which we expect to persist at 1550 nm in the Sagnac measurements.

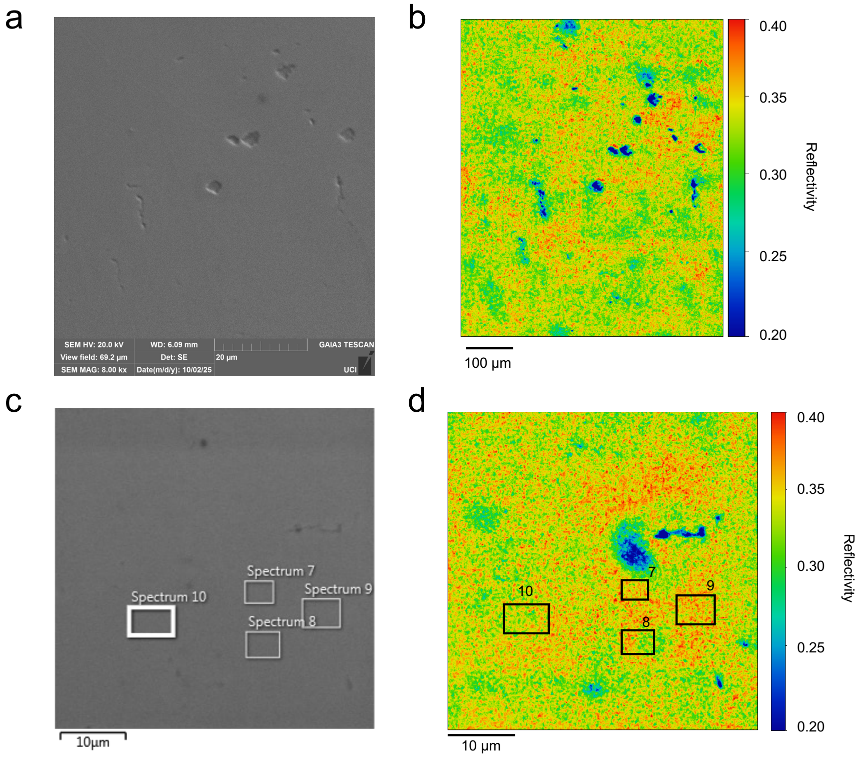

Supplementary Figure 9. Energy-dispersive X-ray spectroscopy (EDX): (a) Electron microscope height image in a large region with several 40 μm -sized pits as landmarks. **(b)** Red-light reflection image (color coded) of the same region as in **(a)**, located using those pits as landmarks, where reflectivity drops to very low levels (blue). Note that even in flat regions, optical reflectivity varies due to Co-composition. **(c)** Electron microscope image of a small and flat region in **(a)**, with Co-composition determined using EDX inside 4 rectangular boxes: box 10: Co 0.292±0.015 > box 8: Co 0.290±0.015 > box 9: Co 0.289±0.015 > box 7: Co 0.286±0.015. **(d)** Red-light reflection image of the same region in **(c)**, showing lower reflectivity in boxes of higher Co composition: box 10: 0.31 ≈ box 8: 0.31 < box 9: 0.33 ≈ box 7: 0.33.

Estimating the Contribution of Magnetization to the MOKE Signal

The conventional mechanism of MOKE (θ_K) is due to the interplay between SOC and band exchange splitting (BES) in the band structure [8] induced by magnetization (M_Z) that is either spontaneous (zero-field) or from an external magnetic field. We can estimate this M_Z-linear contribution to MOKE in our study, which turns out to be extremely small, less than 1%.

This can be seen by comparing the magnetization M_Z (Fig. 2c) and MOKE signal (Fig. 2d, red) during a magnetic field sweep from 0 to 4 T. At zero-field, M_Z (0 T) = 0.01 μ_B, θ_K (0 T) = 195 μrad; at 4 Tesla just below the metamagnetic transition, the magnetization increases to M_Z (4 T) = 0.10 μ_B, and MOKE only changes slightly to θ_K (0 T) = 196 μrad. This indicated that the MOKE due to magnetization is no bigger than (196 -195) μrad / (0.10-0.01 μ_B) = 11 $\mu rad/\mu_B$. Thus, even at 4 Tesla when the magnetization is 0.10 μ_B, the contribution to MOKE from magnetization is at most 11 $\mu rad/\mu_B$ * 0.10 μ_B = 1.1 μrad, i.e. only 0.5 % of the total 196 μrad signal.

Now consider the simultaneous increase of magnetic susceptibility (Fig. 1b) and zero-field MOKE (Fig. 1d). Both reflect the buildup of spin chirality, but one does not cause the other; they are parallel consequences of the same underlying evolution of spin texture.

A similar argument applies to the field sweep across the metamagnetic transition between 4 T and 6 T (Figs. 2c and 2d). In this region, both magnetization and MOKE increase sharply, by 0.08 μ_B and 60 μrad, respectively, due to a sudden reconstruction of the spin configuration (i.e., spin chirality). If the MOKE change were driven primarily by magnetization, it would amount to only 11 $\mu rad/\mu_B$ * 0.08 μ_B = 0.9 μrad, i.e., just 1% of the observed 60 μrad. Therefore, the dominant origin of MOKE is clearly the change in spin chirality, not the net magnetization during the metamagnetic transition.

References:

1. Erskine, J. L. & Stern, E. A. Magneto-optic Kerr Effect in Ni, Co, and Fe. *Phys. Rev. Lett.* **30**, 1329–1332 (1973).
2. Kim, M., Freeman, A. J. & Wu, R. Surface effects and structural dependence of magneto-optical spectra: Ultrathin Co films and CoPt n alloys and multilayers. *Phys. Rev. B* **59**, 9432–9436 (1999).
3. Higo, T. *et al.* Large magneto-optical Kerr effect and imaging of magnetic octupole domains in an antiferromagnetic metal. *Nature Photon* **12**, 73–78 (2018).
4. Kato, Y. D., Okamura, Y., Hirschberger, M., Tokura, Y. & Takahashi, Y. Topological magneto-optical effect from skyrmion lattice. *Nat Commun* **14**, 5416 (2023).
5. Xia, J., Beyersdorf, P. T., Fejer, M. M. & Kapitulnik, A. Modified Sagnac interferometer for high-sensitivity magneto-optic measurements at cryogenic temperatures. *Applied Physics Letters* **89**, 062508 (2006).
6. Feng, Z. *et al.* Nonvolatile Nematic Order Manipulated by Strain and Magnetic Field in a Layered Antiferromagnet. Preprint at https://doi.org/10.48550/arXiv.2507.05486 (2025).
7. Wang, J., Farhang, C., Ortiz, B. R., Wilson, S. D. & Xia, J. Resolving the discrepancy between MOKE measurements at 1550-nm wavelength on kagome metal CsV3Sb5. *Phys. Rev. Mater.* **8**, 014202 (2024).
8. Argyres, P. N. Theory of the Faraday and Kerr Effects in Ferromagnetics. *Phys. Rev.* **97**, 334–345 (1955).
